# Supplementary material for: Post-Exercise Shifts in the Hemato–Biochemical Profile of Unacclimatized Camels (Camelus dromedarius)
Source: Animals (Basel). 2025 Oct 22;15(21):3061. doi: 10.3390/ani15213061 (PMC12608913; doi:10.3390/ani15213061)
Supplement: Supplementary file 1 [file animals-15-03061-s001.zip › Metadata for Dataset (RAW DATA.xlsx).pdf]

Metadata for Dataset (RAW DATA.xlsx)

**Column**	**Description**
Time	Sampling time point (1–6): baseline (PRE), 0 h, 3 h, 6 h, 24 h, and 48 h post-exercise.
Animal	Identifier for each camel (1–7); anonymized unique codes.
RBC	Red blood cell count ($\times 10^6/\mu L$).
Hb	Hemoglobin concentration (g/dL).
Hct	Hematocrit (%).
Bleeding_Time	Bleeding time (minutes).
Osmo	Serum osmolality (mOsm/kg H_2O).
Na	Sodium concentration (mmol/L).
K	Potassium concentration (mmol/L).
Cl	Chloride concentration (mmol/L).
Ca	Calcium concentration (mg/dL).
PO4	Phosphate concentration (mg/dL).
TP	Total protein (g/dL).
Albumin	Serum albumin (g/dL).
Globulin	Calculated globulin (g/dL).
Glucose	Serum glucose (mg/dL).
BUN	Blood urea nitrogen (mg/dL).
Creatinine	Serum creatinine (mg/dL).
AST	Aspartate aminotransferase activity (U/L).
ALT	Alanine aminotransferase activity (U/L).
LDH	Lactate dehydrogenase activity (U/L).
ALP	Alkaline phosphatase activity (U/L).
