# Supplementary material for: Post-Exercise Shifts in the Hemato–Biochemical Profile of Unacclimatized Camels (Camelus dromedarius)
Source: Animals (Basel). 2025 Oct 22;15(21):3061. doi: 10.3390/ani15213061 (PMC12608913; doi:10.3390/ani15213061)
Supplement: Supplementary file 1 [file animals-15-03061-s001.zip › SAS Analysis Code.pdf]

SAS Analysis Code

```
---------------------------------------------------------
  SAS Program for Repeated-Measures ANOVA and Effect Sizes
  Experiment: Exercise in Camels
  Author: Dr. Emad M. Samara
  Software: SAS 9.4
---------------------------------------------------------
```

1. Import dataset (ensure Excel file is in same directory)

```
PROC IMPORT DATAFILE="DATA.xlsx"
   OUT=cameldata
   DBMS=XLSX
   REPLACE;
   SHEET="Sheet1";
RUN;
```

2. Inspect data structure

```
PROC CONTENTS DATA=cameldata; RUN;
PROC MEANS DATA=cameldata N MEAN STD MIN MAX; RUN;
```

3. Repeated-Measures ANOVA for each variable

Time = within-subject factor; Animal = subject (repeated)

```
%macro RepeatedMeasures(var);
   PROC MIXED DATA=cameldata;
      CLASS Animal Time;
      MODEL &var = Time / DDFM=SATTERTHWAITE;
      REPEATED Time / SUBJECT=Animal TYPE=CS R RCORR;
      LSMEANS Time / ADJUST=Tukey PDIFF CL;
      ODS OUTPUT LSMeans=LS&var
            Diffs=Diffs&var
            Tests3=Tests3&var;
      TITLE "Repeated-Measures ANOVA with Tukey Adjustment for &var";
   RUN;
%mend;

%RepeatedMeasures(RBC);
%RepeatedMeasures(Hb);
%RepeatedMeasures(Hct);
%RepeatedMeasures(BleedingTime);
%RepeatedMeasures(Osmo);
%RepeatedMeasures(Na);
%RepeatedMeasures(K);
%RepeatedMeasures(Cl);
%RepeatedMeasures(Ca);
%RepeatedMeasures(PO4);
```

```
%RepeatedMeasures(TP);
%RepeatedMeasures(Albumin);
%RepeatedMeasures(Globulin);
%RepeatedMeasures(Glucose);
%RepeatedMeasures(BUN);
%RepeatedMeasures(Creatinine);
%RepeatedMeasures(AST);
%RepeatedMeasures(ALT);
%RepeatedMeasures(LDH);
%RepeatedMeasures(ALP);
```

4. Combine Tukey-adjusted pairwise results

```
DATA AllTukey;
   SET Diffs:;
RUN;

PROC EXPORT DATA=AllTukey
   OUTFILE="TukeyAdjustedPValues.csv"
   DBMS=CSV
   REPLACE;
RUN;
```

5. Compute Effect Sizes (Cohen's d) for PRE vs. others

```
%macro EffectSizes(var);
   Obtain means and SD for each Time
   PROC MEANS DATA=cameldata NOPRINT;
      CLASS Time;
      VAR &var;
      OUTPUT OUT=Stats&var MEAN=Mean SD=SD N=N;
   RUN;

   Compute Cohen's d comparing each Time vs. PRE (Time=1)
   DATA d&var;
      MERGE Stats&var (RENAME=(Mean=Meani SD=SDi N=Ni))
            Stats&var (RENAME=(Mean=Meanj SD=SDj N=Nj) WHERE=(Time=1));
      BY ALL;
      IF Time NE 1 THEN DO;
         PooledSD = sqrt(((Ni-1)*SDi2 + (Nj-1)*SDj2)/(Ni+Nj-2));
         dvalue  = (Meani - Meanj)/PooledSD;
         95% CI for Cohen's d
         sed = sqrt((Ni + Nj)/(Ni*Nj) + (dvalue2)/(2*(Ni+Nj-2)));
         LowerCI = dvalue - 1.96*sed;
         UpperCI = dvalue + 1.96*sed;
      END;
      KEEP Time dvalue LowerCI UpperCI PooledSD;
   RUN;

   PROC PRINT DATA=d&var LABEL;
      TITLE "Effect Size (Cohen's d) for &var: PRE vs. Other Times";
```

```
    LABEL dvalue="Cohen's d" LowerCI="95% CI Lower" UpperCI="95% CI Upper";
  RUN;

%mend;

%EffectSizes(RBC);
%EffectSizes(Hb);
%EffectSizes(Hct);
%EffectSizes(BleedingTime);
%EffectSizes(Osmo);
%EffectSizes(Na);
%EffectSizes(K);
%EffectSizes(Cl);
%EffectSizes(Ca);
%EffectSizes(PO4);
%EffectSizes(TP);
%EffectSizes(Albumin);
%EffectSizes(Globulin);
%EffectSizes(Glucose);
%EffectSizes(BUN);
%EffectSizes(Creatinine);
%EffectSizes(AST);
%EffectSizes(ALT);
%EffectSizes(LDH);
%EffectSizes(ALP);
```

6. Export Effect Sizes to CSV for documentation

```
DATA Alld;
  SET d:;
RUN;

PROC EXPORT DATA=Alld
  OUTFILE="EffectSizesCohend.csv"
  DBMS=CSV
  REPLACE;
RUN;
```
