# Supplementary material for: Post-Exercise Shifts in the Hemato–Biochemical Profile of Unacclimatized Camels (Camelus dromedarius)
Source: Animals (Basel). 2025 Oct 22;15(21):3061. doi: 10.3390/ani15213061 (PMC12608913; doi:10.3390/ani15213061)
Supplement: Supplementary file 1 [file animals-15-03061-s001.zip › Table S1 (Post-hoc Power Analysis Summary).pdf]

Table S1. Post-hoc Power Analysis Summary

This table summarizes the post-hoc power and precision analysis for all 20 hematological and biochemical variables. Effect sizes (η^2 and Cohen's f) were derived from one-way repeated-measures ANOVA, and observed power was computed using F-test power estimation for six time points (n = 7 animals per time point).

Variable	η^2 (Eta-Squared)	Effect Size f	Observed Power
RBC	0.065	0.263	0.189
Hb	0.056	0.244	0.167
PCV	0.126	0.380	0.380
Bleeding Time	0.865	2.530	1.000
Osmolality	0.505	1.009	0.999
Na^+	0.518	1.037	1.000
K^+	0.504	1.008	0.999
Cl^-	0.060	0.254	0.178
Ca^{2+}	0.348	0.730	0.944
PO_4^{3-}	0.638	1.328	1.000
Total Protein	0.248	0.575	0.766
Albumin	0.266	0.601	0.808
Globulin	0.174	0.459	0.541
Glucose	0.189	0.483	0.591
BUN	0.345	0.726	0.941
Creatinine	0.416	0.844	0.987
AST	0.288	0.637	0.856
ALT	0.119	0.367	0.354
LDH	0.498	0.997	0.999
ALP	0.418	0.847	0.988

Interpretation:

- High statistical power (≥ 0.8) was achieved for 12 variables (e.g., Bleeding Time, Osmolality, Na^+, K^+, PO_4^{3-}, Ca^{2+}, BUN, Creatinine, AST, LDH, ALP), indicating strong sensitivity for medium-to-large effects.
- Moderate power (0.5–0.8) was observed for Total Protein, Albumin, Globulin, and Glucose.
- Lower power (< 0.4) was found for RBC, Hb, Cl^-, and ALT, consistent with their non-significant temporal variation.
