# Supplementary figures and images for: Post-Exercise Shifts in the Hemato–Biochemical Profile of Unacclimatized Camels (Camelus dromedarius)
Source: Animals (Basel). 2025 Oct 22;15(21):3061. doi: 10.3390/ani15213061 (PMC12608913; doi:10.3390/ani15213061)

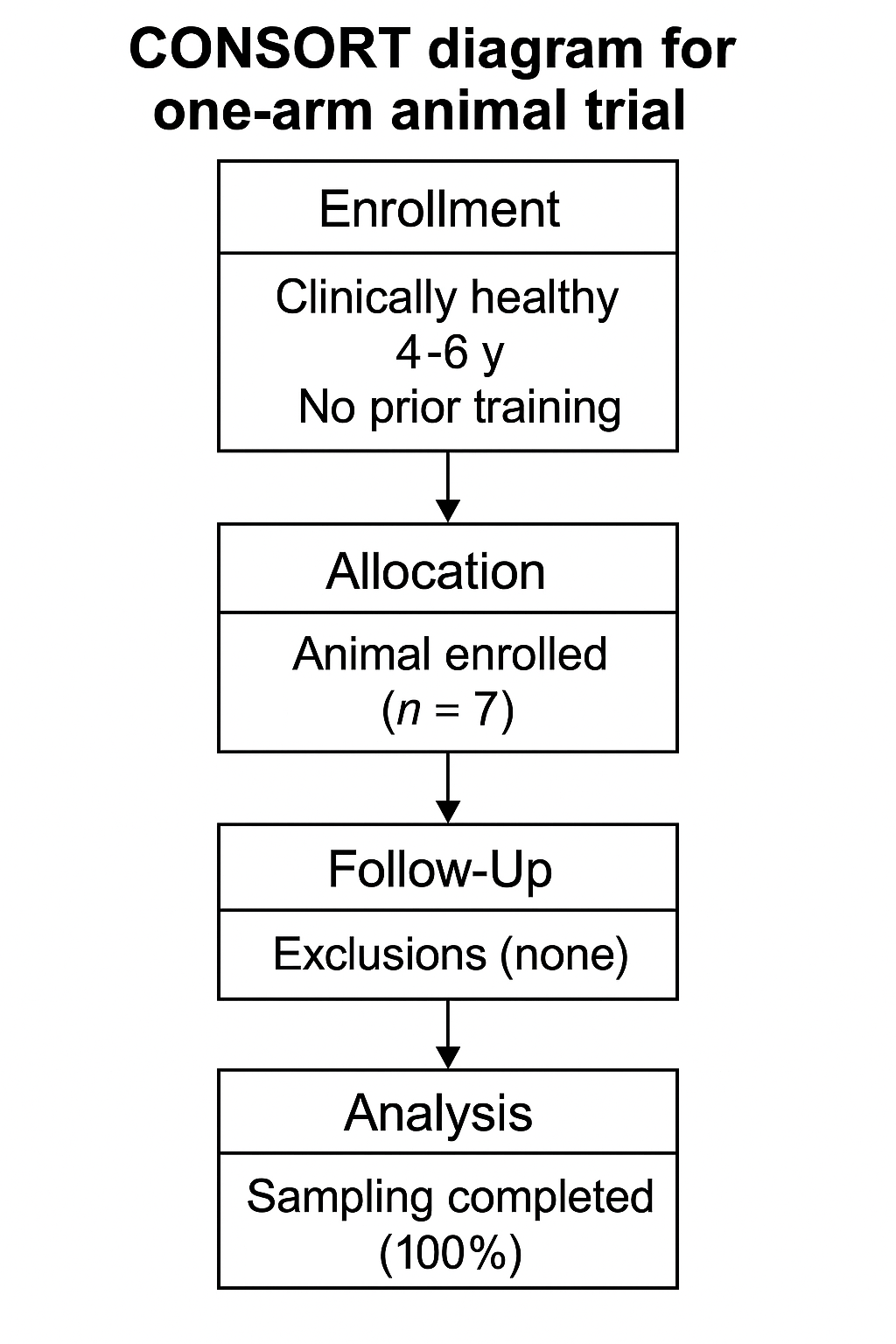

Supplement: Supplementary file 1 [file animals-15-03061-s001.zip › Figure S1 (CONSORT-style flow diagram).png]
